# High-Precision RTT-Based Indoor Positioning System Using RCDN and RPN

**DOI:** 10.3390/s21113701

**Published:** 2021-05-26

**Authors:** Ju-Hyeon Seong, Soo-Hwan Lee, Won-Yeol Kim, Dong-Hoan Seo

**Affiliations:** 1 Department of Liberal Education, Korea Maritime and Ocean University, Busan 49112, Korea; jhseong@kmou.ac.kr; 2 Department of Electrical and Electronics Engineering, Interdisciplinary Major of Maritime AI Convergence, Korea Maritime and Ocean University, Busan 49112, Korea; config5246@naver.com (S.-H.L.); kwy00@naver.com (W.-Y.K.); 3 Division of Electronics and Electrical Information Engineering, Interdisciplinary Major of Maritime AI Convergence, Korea Maritime and Ocean University, Busan 49112, Korea

**Keywords:** indoor positioning, RCDN, RNN, round-trip timing (RTT)

## Abstract

Wi-Fi round-trip timing (RTT) was applied to indoor positioning systems based on distance estimation. RTT has a higher reception instability than the received signal strength indicator (RSSI)-based fingerprint in non-line-of-sight (NLOS) environments with many obstacles, resulting in large positioning errors due to multipath fading. To solve these problems, in this paper, we propose high-precision RTT-based indoor positioning system using an RTT compensation distance network (RCDN) and a region proposal network (RPN). The proposed method consists of a CNN-based RCDN for improving the prediction accuracy and learning rate of the received distances and a recurrent neural network-based RPN for real-time positioning, implemented in an end-to-end manner. The proposed RCDN collects and corrects a stable and reliable distance prediction value from each RTT transmitter by applying a scanning step to increase the reception rate of the TOF-based RTT with unstable reception. In addition, the user location is derived using the fingerprint-based location determination method through the RPN in which division processing is applied to the distances of the RTT corrected in the RCDN using the characteristics of the fast-sampling period.

## 1. Introduction of Indoor Positioning System Methods

With the diversification of embedded communication modules owing to the technological development of smartphones, RTLS (Real-time Locating systems) called IPS (Indoor Positioning Systems) based on such devices were developed. RTLS are a technology that checks or tracks the location of a person or object in the same way as an LBS (Location-based Service) based on a mobile communication network [1,2]. Among them, GPS is widely used as a representative positioning system. However, GPS with very low power transmission creates shadow areas that are difficult to position measurements by obstacles such as buildings and tunnels [3,4,5]. Therefore, various indoor positioning systems were developed to provide LBSs even in indoor areas. Depending on the type of wireless communication, wireless communication-based indoor positioning systems can be broadly divided into fingerprint and time-of-flight (TOF) methods [6,7]. In the former, a user’s location is identified based on the received signal strength indicator (RSSI) [8,9] or channel-state information (CSI) [10,11] depending on the relative distance between the transmitter and the receiver. Wireless communication to which this method can be applied includes Bluetooth [12,13], Wi-Fi [14], and Zigbee [15,16]. Those methods with a positioning resolution of 2–3 m are robust to non-line-of-sight (NLOS) environments because the RSSI measured at all indoor locations at regular intervals is stored as a database (DB) called Radio map and compared with that of the RSSI measured in real-time.

On the other hand, in the TOF method, the distance estimation is based on the transmission/reception time of the radio waves in real-time using chirp-based [17,18] and ultra-wide band (UWB) communications, etc. [19,20,21]. The ranging based on RTT has generally higher accuracy with respect to the RSSI-based Fingerprint method with a positioning resolution of 2–3 m. The positioning accuracy also depends on the technology used for RTT estimation. RTT computed using UWB signals is commonly more accurate with respect to the RTT computed using narrowband signals [22,23,24].

However, a separate AP installation is required according to the communication technology, and with the general TOF method, which is vulnerable to distortion due to reflection or refraction of radio waves, it is difficult to accurately estimate the location in an indoor NLOS environment.

The Wi-Fi-based fingerprint method can be easily applied using a wide infrastructure of smartphones and Wi-Fi access points (APs) and has a high positioning accuracy given that this method considers radio wave attenuation due to obstacles unlike the TOF method. However, the fingerprint method not only has a relatively low position resolution (in meters), but also has a large workload in the training phase to build a DB essential for this system. In particular, as the installation location of the AP changes, the necessary DB should be rebuilt, and security problems due to storage also arise.

To solve this problem, a new Wi-Fi protocol called fine timing measurement (FTM) (or IEEE 802.11.mc standard) was introduced in 2016. To estimate the distance between the transmitter and the receiver, a TOF-based round-trip timing (RTT) approach along with two-way ranging (TWR) was introduced, which can be implemented in a smartphone. The emergence of this protocol means a new indoor location recognition technology based on distance estimation using a smartphone without clock synchronization using Wi-Fi [25,26].

Unlike the Wi-Fi fingerprint method, which requires building an enormous DB called a radio map, this protocol can be easily applied to an indoor positioning system using only the installation coordinate information of the APs. Thus, in the RTT positioning system, trilateration using the relative distances measured via RTT from three or more APs is essential to obtain the user location [27,28,29]. The relative estimated distance between the AP and the smartphone obtained through the RTT has errors ranging from several tens of centimeters to several meters due to the LOS/NLOS environment. Therefore, even a single error in the distance estimated by the RTT due to the characteristics of the trilateration will significantly affect the user location; moreover, the RTT distance and user location estimation errors due to factors such as multipath fading occur more often in an indoor environment [30,31,32,33].

Gentner et al. [34] studied timing-based positioning algorithms, in this case using Wi-Fi-RTT distance estimates. In a typical indoor environment, a Wi-Fi-RTT distance error model was derived. The Wi-Fi-RTT distance error model was included in the likelihood function of a particle filter (PF), and the positioning performance was evaluated in an indoor scenario. These evaluations clearly show the possibility of using Wi-Fi-RTT distance estimates for indoor positioning. Although this study demonstrated the effectiveness of RTT as an indoor location recognition technology, the distance estimation error remains an issue.

Huang et al. [35] proposed a high-precision indoor pedestrian positioning system (HPIPS) based on smartphones. When constructing the training dataset, a fingerprint grayscale image construction method combined with specific AP positions was designed, and the representative physical space features were extracted using a CNN for pedestrian position prediction. Moreover, a particle filter was used for location detection. For direct positioning based on RTT ranging, it is difficult to ensure an accurate estimation of each position, particularly under the influences of NLOS environment and multipath fading.

Cao et al. [30] proposed a Wi-Fi RTT positioning method based on line-of-sight (LOS) identification and range calibration model. The range calibration model for the LOS distance was used to correct the measuring distance, and the scenario information was utilized to constrain the estimated position. However, this system is vulnerable to environmental changes because it is applicable only to environments with a clear scenario and may still exhibit positioning failure.

In these studies, to correct the positioning error of the RTT, which is vulnerable to multipath fading, the models were designed by combining with existing methods or by scenarios. However, it remains difficult to solve the problem of RTT distance errors given the considerable reflections and refractions of radio waves in various indoor environments.

To solve these problems, in this paper, we propose high-precision RTT-based indoor positioning system using an RTT compensation distance network (RCDN) and a region proposal network (RPN). The proposed deep learning-based RCDN and RPN connect the network in an end-to-end manner to accurately measure the user location in real-time through the distance measured from the Wi-Fi RTT. The proposed RCDN collects and corrects a stable and reliable distance prediction value from each RTT transmitter by applying a scanning step to increase the reception rate of the TOF-based RTT with unstable reception. In addition, the user location is derived using the fingerprint-based location determination method through the RPN in which division processing is applied to the RTT-measured distances corrected in the RCDN using the characteristics of the fast-sampling period.

The remainder of this article is organized as follows: Section 2 reviews the Positioning system based on Neural Networks and RTT-based Wi-Fi Positioning; Section 3 presents the proposed fingerprint system; Section 4 shows the Experiment Results and Discussion, and finally, Section 5 presents the conclusions.

## 2. Related Works

### 2.1. Positioning System based on Neural Networks

Neural networks are widely applied as position correction and determination algorithms in positioning systems. Although these objectives are different, a model training phase and a positioning phase are generally essential for a neural network-based positioning system.

The CNN-based indoor positioning system is widely applied to extract features of Wi-Fi DB or RSSI, particularly for data learning model generation in preprocessing, such as in the fingerprint [36,37,38] and TOF [39,40] methods.

In the fingerprint method, the CNN is widely applied to model the pattern of RSSI or CSI measured at each location at regular intervals indoors. Image-based CNN processing is possible by converting an image that combines RSSI or CSI and an indoor map. On the other hand, the TOF is mainly applied to UWB, which enables precise positioning in millimeter units. The actual distances between the transceivers and the measured distances using the TOF method are collected, visualized, and learned using the CNN. Therefore, in the current neural network-based positioning network, the process of collecting actual data and transforming them into data that can be learned by visualizing or preprocessing is indispensable. 

On the other hand, the recurrent neural network (RNN), which is capable of time series processing owing to its repetitive structure, outputs results depending on not only the current input but also historical data. Therefore, RNNs can learn long-term dependencies [39].

**Figure 1:**
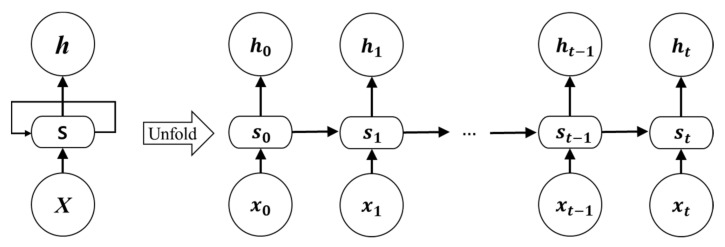
Structure of an RNN.

Figure 1 shows the structure of an RNN. Here, the input to the RNN is *X*, the hidden layer is S, and the output is *h*. Depending on time *t*, the hidden layer S is sequentially connected to have a dependency on the past input *X*.

In the case of an indoor location recognition system, since the characteristics of the RNN are similar to the positioning result having a high correlation in sequence between the user’s previous location and the next location, the tracking accuracy can be improved by applying time-series processing to the RNN.

This RNN-based research is a representative case where long-short term memory (LSTM) and gated recurrent unit (GRU) [41,42,43,44] are applied. These neural network-based methods are mainly studied to improve the location accuracy without distinguishing between fingerprint or TOF.

### 2.2. RTT-Based Wi-Fi Positioning

**Figure 2:**
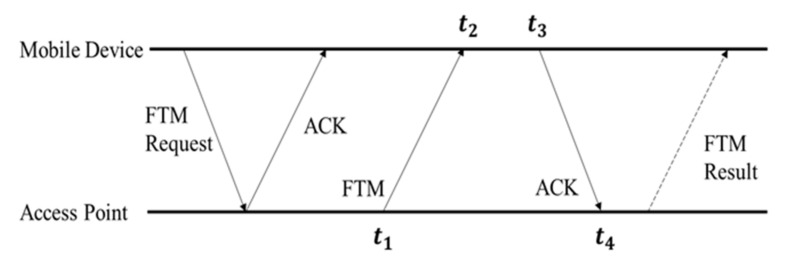
Wi-Fi FTM protocol.

The FTM protocol was newly added to IEEE 802.11 mc, the standard of Wi-Fi in 2016, and includes RTT that estimates the distance between RTT transceivers using the TWR. The RTT is emerging as a new Wi-Fi positioning technology because it can estimate the distance between transceivers, unlike the existing fingerprint method that relies only on the RSSI. Figure 2 shows the details of the FTM protocol, where a mobile device initiates the FTM process by sending an FTM request to an AP. The FTM, a point-to-point (P2P) single-user protocol, involves an exchange of multiple message frames between an AP and a mobile device such as a smartphone or a tablet. The FTM protocol contains five messages, two of which are sent by the initiating device. An AP that supports the FTM protocol responds to the FTM request either to accept or refuse the ranging process. In case of an agreement, the AP starts to send an FTM message and waits for its acknowledgment (ACK) and transmits after the FTM result [30,31,32,33].

The distance between the AP and the mobile device estimated by this method is expressed in (1):

(1) $$\;distance=C\times \frac{\left(t_{4}-t_{1}\right)-\left(t_{3}-t_{2}\right)}{2}$$

Here, C means the velocity of light. Thus, the distance can be estimated through the radio propagation time and C between the two devices. The estimated distance has an error depending on the indoor environment, and research to reduce this error is essential to implementing an accurate positioning system.

## 3. Proposed High-Precision RTT-Based Indoor Positioning System

The Proposed High-Precision RTT-based Indoor Positioning system is designed as a Neural Network that requires learning to reduce RTT errors occurring in indoor environments and to minimize positioning errors. This section gives a specific description of the proposed system designed end-to-end.

### 3.1. Proposed RTT-Based Indoor Positioning System

Generally, the RTT is vulnerable to multipath fading caused by indoor obstacles such as surrounding walls and doors [30,32]. To this end, recent studies stabilized the range through statistical correction or signal path analysis. However, in an actual RTT transmission/reception, various phenomena, such as noise due to the LOS/NLOS environment, measurement errors due to nonreception, and distortion due to signal delay, occur simultaneously.

**Figure 3:**
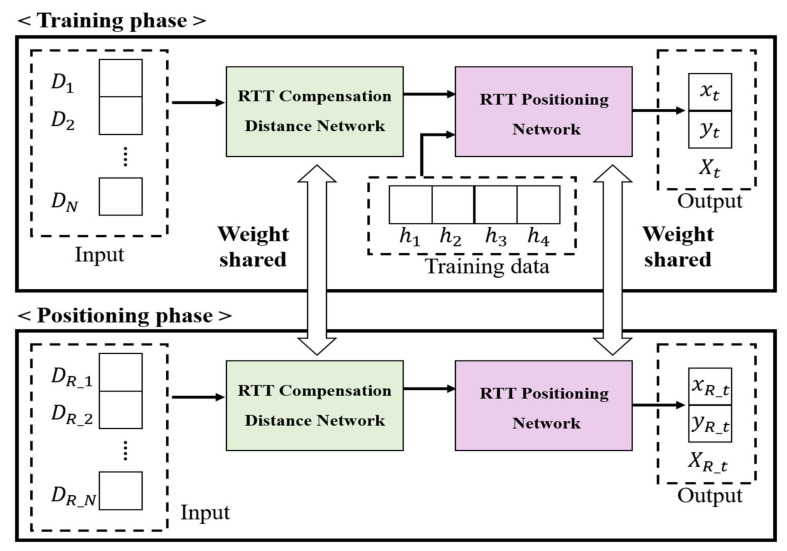
Schematic of proposed system.

To solve this, we propose a high-precision RTT-based indoor positioning system using a compensation network and an RNN. Figure 3 shows the schematic of the proposed high-precision RTT-based indoor positioning system. The proposed network is divided into two parts: an RCDN, which corrects the distance measured by the RTT, and an RPN, which estimates the user’s location based on time-series processing using the corrected distance. The training phase is used as training data by dividing the measured RTT data by $D_{N}$ and $H_{n}$ in a ratio of 8:2. At this time, $H_{n}$ is the data size for 4 APs, and the network is trained using it as an input to the RPN. The weights learned in the training phase are the same network shared with the network in the positioning phase. Then, the positioning phase is used for real indoor positioning in an end-to-end.

In the RCDN, to completely prevent measurement errors due to the RTT distance measurement speed and multipath fading, a scanning step was applied to process data by receiving multiple distance measurements. Thereafter, the CNN is applied, and finally, a compensated distance is outputted.

In the proposed RPN, the distances corrected by the RCDN are simultaneously inputted from three or more APs. Through this, each distance is estimated in the same manner as that via the trilateration, and a user’s location coordinates $X_{t}\in \;\left(x,\;y\right)$ are outputted in real-time.

### 3.2. Proposed RTT Compensation Distance Network (RCDN)

**Figure 4:**
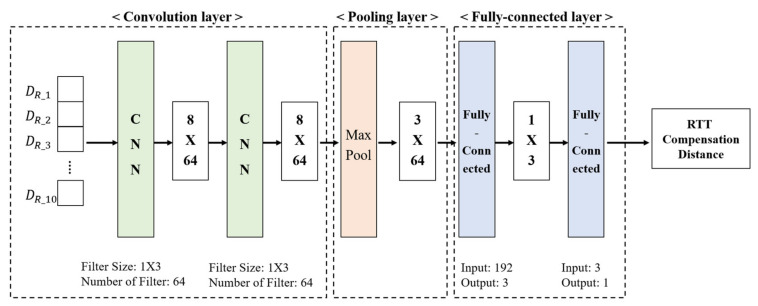
Proposed RTT compensation distance network (RCDN).

Figure 4 shows the proposed RCDN for compensating the Wi-Fi RTT-based measured distances. Here, the input RTT range $D_{R}\in {\mathbb{Z}}^{M}$ is an RTT signal calculated from M APs and is an integer greater than or equal to 0. This $D_{R}\;$ is replaced by preprocessing with negative values due to noise or calculation errors. This network sequentially estimates the corrected distances through a convolution layer, a pooling layer, and a fully connected layer. First, in the convolution layer, a scanning step is applied to the input to completely prevent the measurement error due to the RTT distance measurement speed of 0.02 s and the nonreception situation. The scanning step uses 10 distance values $D_{R\_t1}–D_{R\_t10}$ measured from one AP as the input. The following equation expresses the loss function for learning the proposed RCDN:

(2) $$L_{compensating}\;\left(\hat{D},\bar{D}\;\right)=\frac{\sum_{t=1}^{T}\|\hat{D_{t}}-{\bar{D_{t}}\|}_{2}}{T}$$

where, this function is based on the root-mean-square error (RMSE) corresponding to 10 scanning steps. $\hat{D}$ and $\bar{D}$ denote the estimated and actual distances, respectively. The error distance at the instant of each scanning step T is minimized. The general location information is in a 2D space and follows a Cartesian coordinate system composed of x and y axes. However, the fingerprint method has a problem in classifying multiple classes by separating each location into individual classes. In this multiclass classification problem, there is a limitation in comparing the distance information using a simple numerical position class, and it is unsuitable for the fingerprint method that recognizes patterns in a signal. Therefore, in this study, the location class is one-hot encoded to increase the precision of the location classification process by excluding the relationship between locations. Equation (3) represents the one-hot encoded location information L:

(3) $$L=\left[L_{1},\;L_{2},\ldots ,L_{N-1},\;L_{N}\right],\;L_{N}=\left[0\;or\;1\right]$$

Here, the $L_{n}\in {\left\{0,\;1\right\}}^{N}$ is a sparse vector of 0 or 1 for each point of the grid divided by a certain distance in the positioning target space. This *L* exists as much as *N*, which is the total number of indoor locations set in the proposed system. In the scanning step used to minimize the distance prediction error due to the measurement error, a delay of 0.2 s occurs when 10 pieces of data are received. This hardly affects the user’s distance and location error, and generally, in the case of RSSI reception, a delay of 0.5 s occurs. Since the proposed RDCN predicts the distance value by measuring the RTT distance value in real time, the loss of measured data should be minimized. Therefore, padding, which is commonly applied for data reduction in the convolution layer, was not applied; instead, we designed a filter with a size of 1 × 3 for the convolution layer to prevent data loss.

In addition, to reduce the computational amount of the network and increase the correction accuracy, the number of filters was set to 64 by finetuning the convolution layer. In this network, it is necessary to reduce data to derive multiple input distances into one predicted distance. Here, the pooling layer is applied after the convolution layer to reduce the dimension of the data and prevent overfitting. In the pooling layer, to reduce the spatial size and prevent overfitting, max-pooling with a pooling size of 1 × 2 is applied to a 6 × 64 size feature acquired through two convolution layers.

Finally, two fully connected layers were applied to classify 192 features extracted from the pooling layer into 1 feature. The resulting output is the final distance predicted based on the input 10 distances. Based on the distance that can be measured in an actual environment, values that do not meet the criteria are removed, and finally, a compensated RTT distance is outputted.

### 3.3. Proposed RTT Positioning Network (RPN)

**Figure 5:**
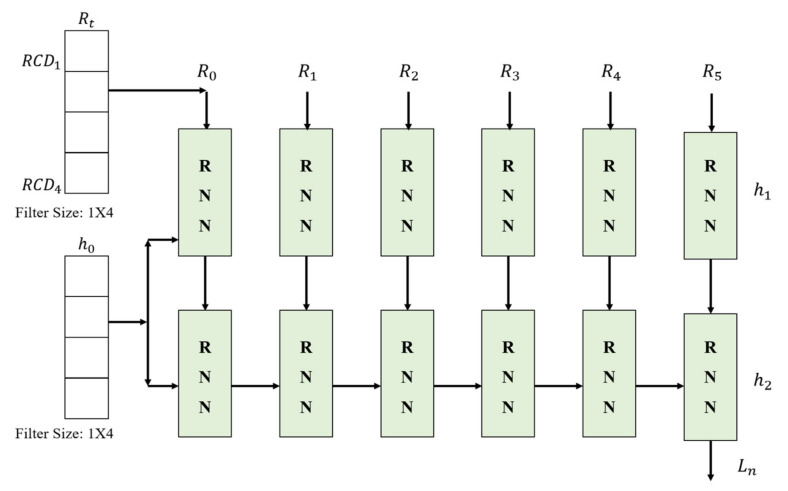
Proposed RNN based positioning network (RPN).

Trilateration, a method for estimating a user’s location using distances in a transmission/reception period, can be applied to estimate locations only when at least three distance measurements are obtained from over three AP receivers. So, TOF-based Trilateration has a limitation on the number of APs, and for this reason, it follows a method based on the k-NN algorithm. This method of fixing the positioning AP not only has a limited range of applications, but also has an upper limit of positioning accuracy. On the other hand, since the proposed RPN receives only the maximum number of APs based on the deep neural network, there is no need to modify the network structure according to the environment. As shown in Figure 5, the corrected distances derived through the RCDN can help estimate the final location of the user in real-time through the RNN-based proposed RPN. $R_{t}$ represents each distance $RCD_{1}–RCD_{4}$ received from four APs. $R_{t}$ measured over time is continuously fed as the input over time, and finally, the user’s location (x, y) is derived. Here, the location errors are corrected by sequentially inputting and processing the measured distances into five RNN networks. The hidden layer $h_{n}\in {\mathbb{R}}^{N\times 1}$ inputs the user’s location in each RNN to the next network. Since the proposed RPN is composed of two RNN layers, it is composed of $h_{1}$ and $h_{2}$. This is calculated as the real-time position $L_{n}$. The user locations at this time are derived again. The internal structure of the proposed RPN combines the input and hidden values and computes them through the fully connected layer.

In general, the RNN, which is widely employed for time series data analysis, has a classic XOR problem in its fully connected network because it is composed of a single layer given its high complexity. In particular, the problem of regressing distances and mapping them to coordinates, such as RTT, inevitably has an XOR structure in which the classification objects intersect. 

Therefore, to solve this problem, two RNNs are used in the proposed RPN. In a general positioning environment, the user’s position does not change rapidly within 1 s, which is the RTT collection time for positioning. Therefore, the final user’s locations are estimated through many-to-one method rather than a many-to-many method that outputs results for each node of the RNN. The coordinates finally derived are the user’s locations, and real-time tracking is possible based on the network characteristics.

When two networks are connected in series, the learning efficiency is good because having one loss function lowers the learning complexity. However, since the RTT signal changes in different environments depending on the arrangement structure and the noise is large, designing a single loss function based on the final positioning result causes an overfitting problem for an individual environment. The proposed system adds the result of the RCDN to the loss function so that this network can be applied equally to other APs rather than using a weight map suitable for a specific AP. Equation (4) expresses the loss function L of the entire network for this purpose:

(4) $$L_{positioning}=-\sum_{t=1}^{T}\bar{L_{t}}\log\hat{L_{t}}$$

(5) $$L=L_{compensating}+L_{positioning}$$

Here, $L_{compensating}$ and $L_{positioning}$ represent the loss functions of the RCDN and RPN (cross-entropy), respectively. Each loss function is summed to derive the loss function of the final system, and through this, it is possible to design a system structure capable of end-to-end learning.

## 4. Experiment Results and Discussion

### 4.1. Experimental Environment and RTT Configuration

We obtained the RTT distance using WILD [32,33], a product with a built-in Intel AC8260 Wi-Fi module produced by CompuLab that can implement RTT. The transceiver mode was set for Wi-Fi through the FTM management command, and the change channel bandwidth was unified to 80 MHz. We set 2.4 GHz as the change band, and it was possible to measure up to IEEE 802.11ac. Finally, the proposed network design and trial were directly implemented using Python.

**Figure 6:**
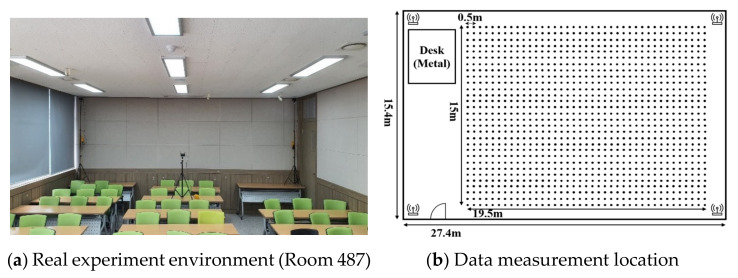
Experimental environment of proposed system.

To construct basic deep neural networks, it is essential to acquire data for learning. Figure 6 shows the experimental environment of the proposed network. As shown (a), four RTT APs were installed in Room 487, College of Engineering Building No. 1 of Korea Maritime and Ocean University to secure RTT data. The experimental environment includes 25 desks 0.72 m high and 3 chairs each. Also, on the opposite side of (a) there is a 1.6 m high iron tabletop. As shown (b), The experiment was measured 200 times per location at 0.5 m intervals, and a total of 1170 location points were measured. Through this data collection process, learning data and real-time positioning data were classified.

Through this RTT data collection process, the data set based on the built database is divided into training, cell, and experimental data in a general ratio of 6.4:1.6:2, respectively. The data were normalized min–max from the minimum distance of 1–20 m. This is a data value considering the error in the maximum and minimum distance between the actual transmitter and the receiver, and all measured data have an integer value between 100 and 2000 in cm. In addition, overfitting was prevented by separating the training data and verification data in advance during the training process of the network.

### 4.2. Learning Results of Proposed Positioning Network

The proposed high-precision RTT-based indoor positioning system was learned by combining a compensation network and a positioning network in an end-to-end manner, making it easy to apply to positioning systems by processing several processes at once.

Since the proposed positioning network is structurally in the form of a classification network, it has accuracy for one-hot encoded positions. Equation (6) shows the accuracy of the entire network:

(6) $$Accuracy=\frac{TP+FN}{Total}$$

here, TP and FN indicate the number of cases where the position L is the correct answer and the case where the position L is incorrect. Since this indicates only the selection of the correct point, the positional accuracy cannot be adequately expressed. Therefore, by mapping the estimated position to the coordinate *X*, the error between the prediction and the actual is calculated as in Equation (7), which is RMSE:

(7) $$RMSE=\sum_{t=0}^{T}\frac{\|\hat{X_{t}}-{\bar{X_{t}}\|}_{2}}{T}$$

where, $X_{t}$ refers to the position coordinate estimated at time T, and calculates the average over the entire time T. In addition, the proposed positioning system includes a correction network for the RTT signal.

(8) $$\;Ranging\;error=\frac{1}{M}\sum_{m=0}^{M}\sum_{t=1}^{T}\frac{\left|\hat{D_{t,m}}-\bar{D_{t,m}}\right|}{T}$$

Here, $\hat{D_{t,m}}$ and $\bar{D_{t,m}}$ represent the distance of the m-th AP at point t.

**Figure 7:**
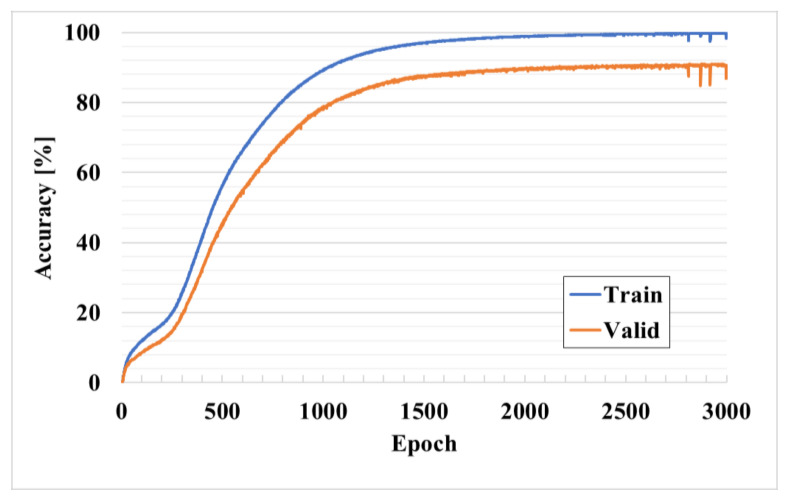
Accuracy results of training and verification data with respect to epoch.

Figure 7 shows the accuracy results of the training and verification data with respect to the epoch. The x-axis represents the epoch, which is the number of learning completions for the entire dataset, and the y-axis represents the positioning accuracy. The experiment was carried out for up to 3000 epochs, at which point the network converged. Here, the blue and orange colors represent the learning and verification data, respectively. After 3000 training iterations, the training data converge to more than 99% and the verification data to more than 90%. The learning rate declines from 250 iterations and then rises again. This is the tuning that occurs in the learning process of the connected RPN after completing the learning of a relatively simple problem by the RCDN owing to the end-to-end frame.

**Figure 8:**
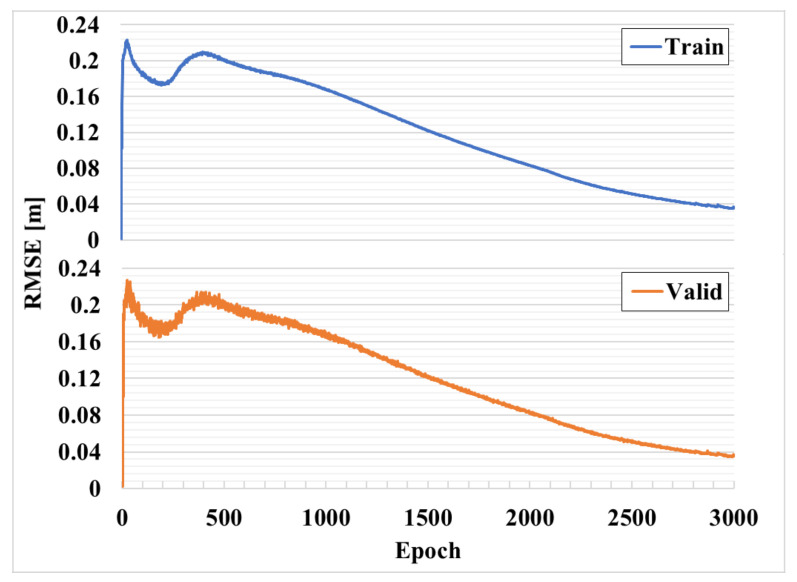
RMSE corresponding to learning of compensation network for 3000 epochs.

Figure 8 shows the RMSE corresponding to the learning of the compensation network for 3000 epochs. Through this, it is possible to analyze in detail the curve generated in the 250th epoch, shown in Figure 7, and to analyze the learning process of the compensation network. The x-axis represents the epoch, and the y-axis represents the RMSE, which is a measure to check the difference between the estimated value or the value predicted by the model and the value observed in the actual environment. Here, unlike the accuracy result shown in Figure 7, the result is more accurate as the RMSE decreases. In this Figure, it has a characteristic shape at approximately 250 epochs as in Figure 7. The CNN-based RCDN that compensates for the distance result of the RTT converges at a fast rate in 10 epochs or less and has a result close to zero. However, since the loss function is shared with the RPN, the RMSE momentarily increases and converges as the learning progresses. As confirmed in previous studies, the RTT distance value is noisy, and since the variance is large, the data averaged over 10 times are also unstable, and a difference from the actual distance value is observed. Therefore, since the auxiliary distance data for learning are the measured RTT distance values, not the distance values obtained through actual surveying, the RCDN relearns by matching with the actual position.

A typical RTT signal is vulnerable to multipath fading, and thus has a different noise distribution depending on the installation location of the AP, which is equally applied to the RDCN. Because the statistical approach does not take this part into account, the APs have different error distributions. However, the proposed RCDN has the same error distribution because it normalizes the signals of all APs in the same form through learning.

**Figure 9:**
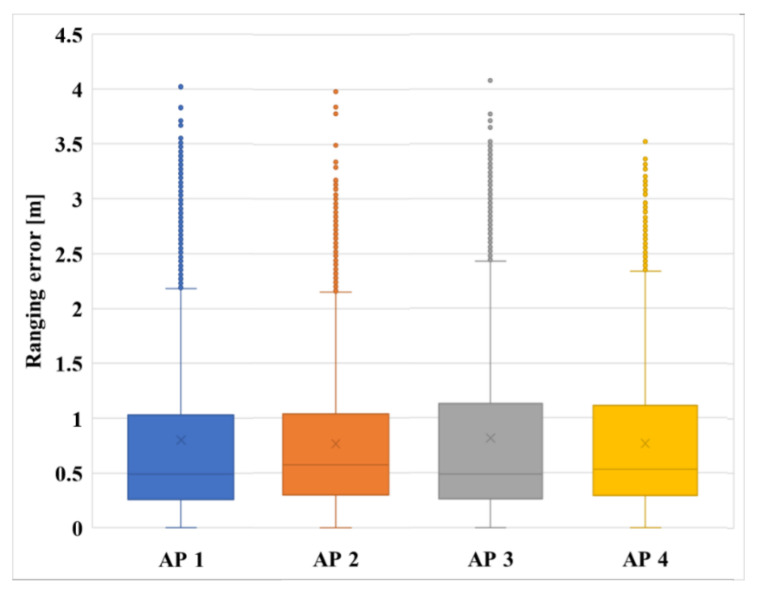
RTT distance errors of each AP normalized by the RCDN.

Figure 9 shows the RTT distance error of each AP normalized by the RCDN. The x-axis represents each AP, and the y-axis represents the distance error. Here, all the APs have an average error of 0.5 m and an error range within a maximum of 4 m. Since the proposed positioning network is designed with the user’s location determined via fingerprint technology, it can be predicted that an average error of 0.5 m represents a higher position resolution and higher accuracy compared to that of the Wi-Fi fingerprint method, which has an error range of 2–3 m.

### 4.3. Positioning Performance Evaluation

To verify the accuracy of the proposed positioning network, the estimated distance errors and user location errors with respect to the training strategy were analyzed. Table 1 shows the RTT distance estimation error with respect to the training strategy. The result of filtering through the standard score that finds outliers through signal variance is 0.93 m, the result of independently learning only the proposed RCDN is 0.81 m, and the result of learning by integrating with the proposed RPN (end-to-end) is rewarded as 0.60 m. Because the reward network tracks only the results of the training data, the result is at the same level as the standard score. However, when the proposed network is trained through an end-to-end training strategy, the measured distance is compensated as close as possible to the actual distance because it is recalibrated through location information.

**Figure 10:**
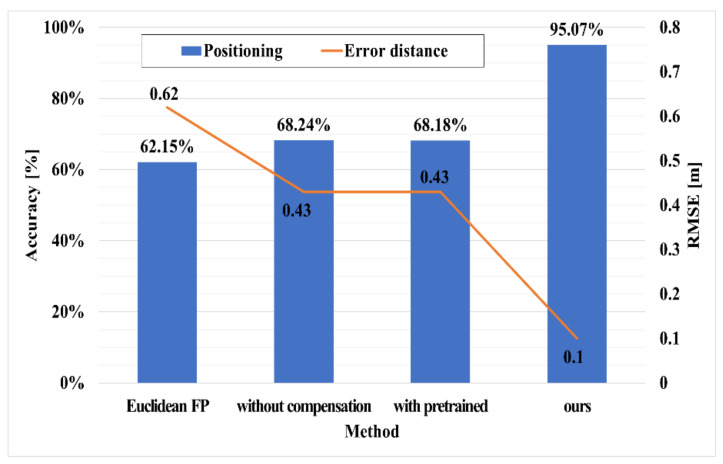
Accuracy and RMSE results according to each network frame.

Figure 10 compares the actual positioning performance by combining the results of Table 1 with the positioning algorithm. The x-axis represents the case of adapting the Euclidean distance using measured data, the case of learning only the RPN, the case of adapting the non-end-to-end, and the case of adapting the proposed system.

In the case of using the Euclidean distance, since the error distance is used without correction, it has an accuracy of 60% and an RMSE of 0.6 m. The case applying only RPN and non-end-to-end show almost the same performance improvement. In the case of applying only RPN, the measured RTT distance is corrected, and in the case of non-end-to-end, the performance is slightly improved because complete fitting is not performed by individual learning.

The case of end-to-end as the proposed system has a positioning classification accuracy of more than 90% and a positioning error of approximately 0.1 m.

Table 2 shows the positioning accuracies of the positioning algorithms. To verify the positioning accuracy, least squares [37], Euclidean distance [38], and MIMO [45], which are representatively applied in the latest studies, were compared with the proposed network. For the positioning error, the average error was derived based on the outputted final location coordinates. The error is 0.62 m when Euclidean distance, which is typically used in the fingerprint method, is applied as it is, whereas the proposed RTT positioning minimizes the position error to 0.10 m in real-time by applying distance correction and time-series processing through a deep neural network.

**Figure 11:**
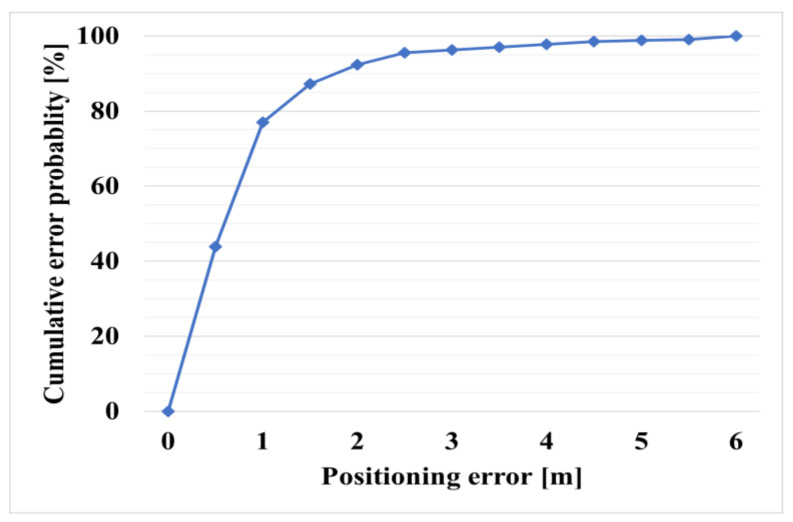
Cumulative distribution function (CDF) of positioning error in case of proposed system.

Figure 11 shows the cumulative distribution function (CDF) of the positioning error. The CDF is a function representing the probability that a given random variable is less than or equal to a specific value. The x-axis represents the positioning error, and the y-axis represents the cumulative error probability. The result shows that the error is less than 2 m; therefore, the stability of the positioning accuracy and error magnitude is confirmed through Table 2 and Figure 11. 

**Figure 12:**
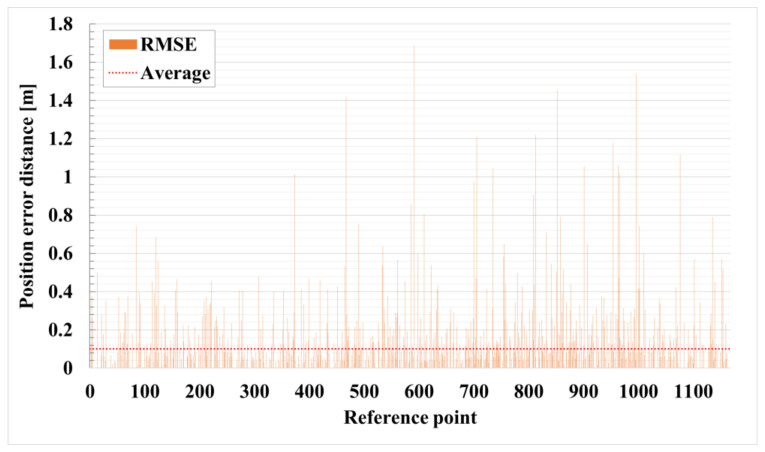
Positioning error distance for each reference point in the case of the proposed system.

Figure 12 shows the positioning error distance according to each reference point. The reference points of the x-axis were assigned sequentially starting from the upper right of Figure 6. The y-axis is the accuracy derived through Equation (6). The average accuracy indicated by the red dotted line is 0.10 m. Most of the results are distributed near the mean, but the index of the reference point close to the wall increases the error of the signal by 30 units. This is because the RTT signal becomes unstable due to the wall surface. Also, the closer to the area with a large index, which is an area close to a metal object, the greater the error of the signal.

**Figure 13:**
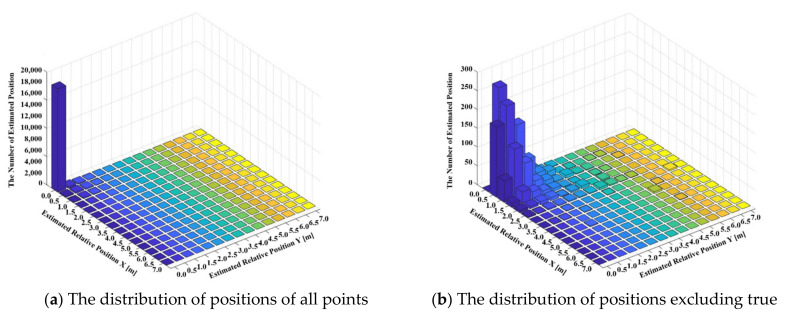
Estimated position distribution relative to ground truth.

Figure 13 shows the relative distribution of the estimated location based on the actual location. In other words, this means how well the proposed network estimates the actual location of (0,0). The experimental results have an x-axis and a y-axis of the first quadrant because the absolute value is taken to increase visibility. (a) shows all estimated positions, and since the estimation frequency of (0,0) is absolutely high, (b) shows the result excluding the origin, which is the correct answer, to improve readability. Most of the results of about 18,000 are located at the origin, with relatively low frequency at the rest of the positions. In the result of (b) excluding the origin, it can be confirmed that most of the estimation occurs within 2 m from the origin. Through this, the accuracy of the proposed positioning system can be confirmed.

## 5. Conclusions

Wi-Fi RTT, which is an IEEE 802.11.mc standard, is a distance estimation technology based on TWR and is being extensively studied for indoor positioning. However, it is difficult to accurately detect the location when no signal is received, and a large distance error occurs due to multipath fading. Therefore, in this study, we developed a high-precision RTT-based indoor positioning system using an RCDN and RPN. The RCDN designed based on an end-to-end framework employs a scanning step for a stable distance prediction and corrects the distance measured during the RTT transceiver. Subsequently, the designed RPN accurately estimates the user location by grafting the distances corrected through the RCDN into the fingerprint method in time-series processing through the RNN.

The results of experiments conducted to verify the proposed system showed that the proposed system can locate a user’s indoor location in real-time in an end-to-end manner. The different inherent errors of each AP were corrected almost identically through the RCDN, and the average error was reduced to approximately 0.6 m from the measured distance. The RPN with the fingerprint positioning method at intervals of 0.5 m corrected the distance errors of each AP through time series processing, confirming a high position accuracy of 0.1 m. The proposed system simplifies the application of an indoor location recognition system based on deep learning through a combination of networks and enables user location tracking and highly precise positioning through time series processing.

In the future, based on this work, we intend to study a network system applicable to more complex environments by designing an AP-centered correction network rather than a spatial-centered correction network.

## Figures and Tables

**Table 1:** Estimated RTT distance errors with respect to each compensation methods.

Compensation Method	Standard Score	Only RCDN	Proposed Network (End-to-End)
Ranging error (m)	0.93	0.81	0.60

**Table 2:** Positioning accuracies of positioning algorithm.

Method	Least Squares [37]	Euclidean Distance [38]	MIMO [45]	Proposed System
Accuracy (m)	3.09	0.62	0.43	0.10

## Data Availability

Not applicable.

## References

[1] Jiménez A.R., Seco F. (2021). Improving the Accuracy of Decawave’s UWB MDEK1001 Location System by Gaining Access to Multiple Ranges. Sensors.

[2] Frankó A., Vida G., Varga P. (2020). Reliable identification schemes for asset and production tracking in industry 4.0. Sensors.

[3] Jiang C., Xu B., Hsu L.T. (2021). Probabilistic approach to detect and correct GNSS NLOS signals using an augmented state vector in the extended Kalman filter. GPS Solut..

[4] Pardhasaradhi B., Srihari P., Aparna P. (2021). Navigation in GPS Spoofed Environment Using M-Best Positioning Algorithm and Data Association. IEEE Access.

[5] Xiang C., Zhang S., Xu S., Alexandropoulos G.C. (2021). Self-Calibrating Indoor Localization with Crowdsourcing Fingerprints and Transfer Learning. arXiv Prepr..

[6] Alhomayani F., Mahoor M.H. (2020). Deep learning methods for fingerprint-based indoor positioning: A review. J. Locat. Based Serv..

[7] Seong J.H., Seo D.H. (2019). Wi-Fi fingerprint using radio map model based on MDLP and Euclidean distance based on the Chi squared test. Wirel. Netw..

[8] Ssekidde P., Steven Eyobu O., Han D.S., Oyana T.J. (2021). Augmented CWT Features for Deep Learning-Based Indoor Localization Using WiFi RSSI Data. Appl. Sci..

[9] Sun H., Zhu X., Liu Y., Liu W. (2020). Construction of Hybrid Dual Radio Frequency RSSI (HDRF-RSSI) Fingerprint Database and Indoor Location Method. Sensors.

[10] Wang J., Park J.G. An enhanced indoor ranging method using CSI measurements with Extended Kalman filter. Proceedings of the 2020 IEEE/ION Position, Location and Navigation Symposium (PLANS).

[11] Dang X., Tang X., Hao Z., Ren J. (2020). Discrete Hopfield neural network based indoor Wi-Fi localization using CSI. EURASIP J. Wirel. Commun. Netw..

[12] Bai L., Ciravegna F., Bond R., Mulvenna M. (2020). A low cost indoor positioning system using Bluetooth low energy. IEEE Access.

[13] Ho Y.H., Chan H.C. (2020). Decentralized adaptive indoor positioning protocol using Bluetooth Low Energy. Comput. Commun..

[14] Seong J.H., Lee S.H., Yoon K.K., Seo D.H. (2019). Ellipse coefficient map-based geomagnetic fingerprint considering azimuth angles. Symmetry.

[15] Uradzinski M., Guo H., Liu X., Yu M. (2017). Advanced indoor positioning using zigbee wireless technology. Wirel. Pers. Commun..

[16] Zhen J., Liu B., Wang Y., Liu Y. (2020). An improved method for indoor positioning based on ZigBee technique. Int. J. Embed. Syst..

[17] Yang Y., Wang M., Qiao Y., Zhang B., Yang H. (2020). Efficient marginalized particle smoother for indoor CSS–TOF localization with non-Gaussian errors. Remote Sens..

[18] An Z., Lin Q., Yang L., Guo Y. (2020). Revitalizing Ultrasonic Positioning Systems for Ultrasound-Incapable Smart Devices. IEEE Trans. Mob. Comput..

[19] Feng D., Wang C., He C., Zhuang Y., Xia X.G. (2020). Kalman-filter-based integration of IMU and UWB for high-accuracy indoor positioning and navigation. IEEE Internet Things J..

[20] Zhang Y., Duan L. (2020). Toward elderly care: A phase-difference-of-arrival assisted ultra-wideband positioning method in smart home. IEEE Access.

[21] Zhang H., Zhang Z., Gao N., Xiao Y., Meng Z., Li Z. (2020). Cost-effective wearable indoor localization and motion analysis via the integration of UWB and IMU. Sensors.

[22] De Angelis G., Moschitta A., Carbone P. (2016). Positioning techniques in indoor environments based on stochastic modeling of UWB round-trip-time measurements. IEEE Trans. Intell. Transp. Syst..

[23] Martinelli A., Jayousi S., Caputo S., Mucchi L. UWB positioning for industrial applications: The galvanic plating case study. Proceedings of the 2019 International Conference on Indoor Positioning and Indoor Navigation (IPIN).

[24] Martinelli A., Dolfi M., Morosi S., Mucchi L., Paoli M., Agili A. (2020). Ultra-wide Band Positioning in Sport: How the Relative Height Between the Transmitting and the Receiving Antenna Affects the System Performance. Int. J. Wirel. Inf. Netw..

[25] Sun M., Wang Y., Xu S., Qi H., Hu X. (2020). Indoor positioning tightly coupled Wi-Fi FTM ranging and PDR based on the extended Kalman filter for smartphones. IEEE Access.

[26] Shao W., Luo H., Zhao F., Tian H., Yan S., Crivello A. (2020). Accurate indoor positioning using temporal-spatial constraints based on Wi-Fi fine time measurements. IEEE Internet Things J..

[27] Fang X., Chen L. (2020). An optimal multi-channel trilateration localization algorithm by radio-multipath multi-objective evolution in RSS-ranging-based wireless sensor networks. Sensors.

[28] Yang B., Guo L., Guo R., Zhao M., Zhao T. (2020). A novel trilateration algorithm for RSSI-based indoor localization. IEEE Sens. J..

[29] Shi Y., Shi W., Liu X., Xiao X. (2020). An RSSI Classification and Tracing Algorithm to Improve Trilateration-Based Positioning. Sensors.

[30] Cao H., Wang Y., Bi J., Xu S., Si M., Qi H. (2020). Indoor Positioning Method Using WiFi RTT Based on LOS Identification and Range Calibration. ISPRS Int. J. Geo-Inf..

[31] Ma C., Wu B., Poslad S., Selviah D.R. (2020). Wi-Fi RTT Ranging Performance Characterization and Positioning System Design. IEEE Trans. Mob. Comput..

[32] Markus B., Toni F., Frank E., Markus E., Frank D., Marcin G. (2020). Comparison of 2.4 GHz WiFi FTM- and RSSI-Based Indoor Positioning Methods in Realistic Scenarios. Sensors.

[33] Horn B.K. (2020). Doubling the Accuracy of Indoor Positioning: Frequency Diversity. Sensors.

[34] Gentner C., Ulmschneider M., Kuehner I., Dammann A. WiFi-RTT Indoor Positioning. Proceedings of the 2020 IEEE/ION Position, Location and Navigation Symposium (PLANS).

[35] Huang L., Yu B., Li H., Zhang H., Li S., Zhu R., Li Y. (2020). HPIPS: A high-precision indoor pedestrian positioning system fusing WiFi-RTT, MEMS, and map information. Sensors.

[36] Seong J.H., Seo D.H. (2020). Selective unsupervised learning-based Wi-Fi fingerprint system using autoencoder and GAN. IEEE Internet Things J..

[37] Hsieh C.H., Chen J.Y., Nien B.H. (2019). Deep learning-based indoor localization using received signal strength and channel state information. IEEE Access.

[38] Wang X., Wang X., Mao S. (2018). Deep convolutional neural networks for indoor localization with CSI images. IEEE Trans. Netw. Sci. Eng..

[39] Cui Z., Gao Y., Hu J., Tian S., Cheng J. (2020). LOS/NLOS identification for indoor UWB positioning based on Morlet wavelet transform and convolutional neural networks. IEEE Commun. Lett..

[40] Nguyen D.T.A., Lee H.G., Jeong E.R., Lee H.L., Joung J. (2020). Deep learning-based localization for UWB systems. Electronics.

[41] Zhang Y., Xiong R., He H., Pecht M.G. (2018). Long short-term memory recurrent neural network for remaining useful life prediction of lithium-ion batteries. IEEE Trans. Veh. Technol..

[42] Wu L., Chen C.H., Zhang Q. (2019). A mobile positioning method based on deep learning techniques. Electronics.

[43] Tarekegn G.B., Juang R.T., Lin H.P., Adege A.B., Munaye Y.Y. (2020). DFOPS: Deep learning-based fingerprinting outdoor positioning scheme in hybrid networks. IEEE Internet Things J..

[44] Sun H., Zhu X., Liu Y., Liu W. (2020). WiFi based fingerprinting positioning based on Seq2seq model. Sensors.

[45] Hoang M.T., Yuen B., Dong X., Lu T., Westendorp R., Reddy K. (2019). Recurrent neural networks for accurate RSSI indoor localization. IEEE Internet Things J..

